# Emerging roles of hydrogen sulfide-metabolizing enzymes in cancer

**DOI:** 10.1080/13510002.2024.2437338

**Published:** 2024-12-06

**Authors:** Alyaa Dawoud, Rana A. Youness, Kareem Elsayed, Heba Nafae, Hoda Allam, Hager Adel Saad, Carole Bourquin, Csaba Szabo, Reham Abdel-Kader, Mohamed Z. Gad

**Affiliations:** a Biochemistry Department, Faculty of Pharmacy and Biotechnology, German University in Cairo (GUC), New Cairo, Egypt; b School of Medicine, University of North Carolina, Chapel Hill, NC, USA; c Molecular Biology and Biochemistry Department, Faculty of Biotechnology, German International University, Cairo, Egypt; d Biochemistry Department, Faculty of Biotechnology, October University for Modern Sciences and Arts (MSA), Giza, Egypt; e School of Pharmaceutical Sciences, Institute of Pharmaceutical Sciences of Western Switzerland, Department of Anaesthesiology, Pharmacology, Intensive Care and Emergency Medicine, University of Geneva, Geneva, Switzerland; f Chair of Pharmacology, Section of Science and Medicine, University of Fribourg, Fribourg, Switzerland; g Pharmacology and Toxicology Department, Faculty of Pharmacy and Biotechnology, German University in Cairo (GUC), New Cairo, Egypt

**Keywords:** H_2_S, metabolism, cysteine aminotransferase (CAT), sulfide quinone oxidoreductase (SQOR), ethylmalonic encephalopathy protein 1 (ETHE1), sulfite oxidase (SUOX)

## Abstract

Gasotransmitters play crucial roles in regulating many physiological processes, including cell signaling, cellular proliferation, angiogenesis, mitochondrial function, antioxidant production, nervous system functions and immune responses. Hydrogen sulfide (H_2_S) is the most recently identified gasotransmitter, which is characterized by its biphasic behavior. At low concentrations, H_2_S promotes cellular bioenergetics, whereas at high concentrations, it can exert cytotoxic effects. Cystathionine β-synthetase (CBS), cystathionine-γ-lyase (CSE), 3-mercaptopyruvate sulfurtransferase (3-MST), and cysteinyl-tRNA synthetase 2 (CARS2) are pivotal players in H_2_S biosynthesis in mammalian cells and tissues. The focus of this review is the regulation of the various pathways involved in H_2_S metabolism in various forms of cancer. Key enzymes in this process include the sulfide oxidation unit (SOU), which includes sulfide:quinone oxidoreductase (SQOR), human ethylmalonic encephalopathy protein 1 (hETHE1), rhodanese, sulfite oxidase (SUOX/SO), and cytochrome c oxidase (CcO) enzymes. Furthermore, the potential role of H_2_S methylation processes mediated by thiol S-methyltransferase (TMT) and thioether S-methyltransferase (TEMT) is outlined in cancer biology, with potential opportunities for targeting them for clinical translation. In order to understand the role of H_2_S in oncogenesis and tumor progression, one must appreciate the intricate interplay between H_2_S-synthesizing and H_2_S-catabolizing enzymes.

## 1. Introduction

Nitric oxide (NO), carbon monoxide (CO), and hydrogen sulfide (H2S) are the three small, diffusible, labile gaseous molecules known as ‘Gasotransmitters’ [1]. They are endogenously produced inside our body. They orchestrate an array of molecular pathways in normal physiological and pathological conditions [2].

The introduction of the ‘gasotransmitters concept’ into the molecular pathophysiology of several diseases has overturned a lot of the conventional perceptions about intracellular communication. Being a gaseous molecule, this makes the concept of storage in vesicular structures impossible and therefore must be re-synthesized whenever needed [3]. This signifies the importance of the synthesizing machinery of those gasotransmitters and their nuanced regulatory system [3]. Nonetheless, gasotransmitters instead of binding to extracellular receptors as any other signaling molecule, they can easily diffuse between cells to interact with their targets, thus highlighting their versatile ability to affect multiple signaling cascades simultaneously [4].

Mammalian Hydrogen sulfide (H_2_S) generation was first detected in liver homogenates by Binkley and de Vigneaud in 1942 [5]. Several subsequent studies reported the presence of H_2_S in mammalian cells and tissues, including human postmortem brain tissues [6–8]. While H_2_S is conventionally associated with toxic effects, it was later realized that when endogenously generated and present at lower concentrations, it can also play important physiological regulatory roles in the body [6]. It can act as a vasodilator, improving blood flow and reducing blood pressure [9]. Additionally, H_2_S can reduce free radical-mediated tissue damage, either by directly interacting with free radicals or oxidants or upregulating various antioxidant enzyme systems [10,11]. Subsequent work demonstrated that H_2_S also regulates multiple receptor-mediated mechanisms and cellular signaling cascades [12–17]. Part of the signaling function of gasotransmitters (including H_2_S) is dependent on their chemical reactivity with oxygen and metals [4,18–21]. Additionally, gasotransmitters, including H_2_S, can function through various second-messenger systems, such as the cyclic guanosine monophosphate (cGMP) and cyclic adenosine monophosphate (cAMP) systems, often in a cooperative manner [3,14,22]. Moreover, H_2_S – and closely related species such as reactive polysulfides – also affect membrane-bound or intracellular proteins by chemically modifying the sulfhydryl or thiol groups (-SH) of cysteine amino acids [3,14,23]. In this process, the sulfhydryl group (-SH) changes into a persulfide (-SSH) group through a reaction termed sulfhydration or persulfidation. This modification, in some cases (but not always) can alter the catalytic activity of the affected protein [24,25].

**Figure 1.**
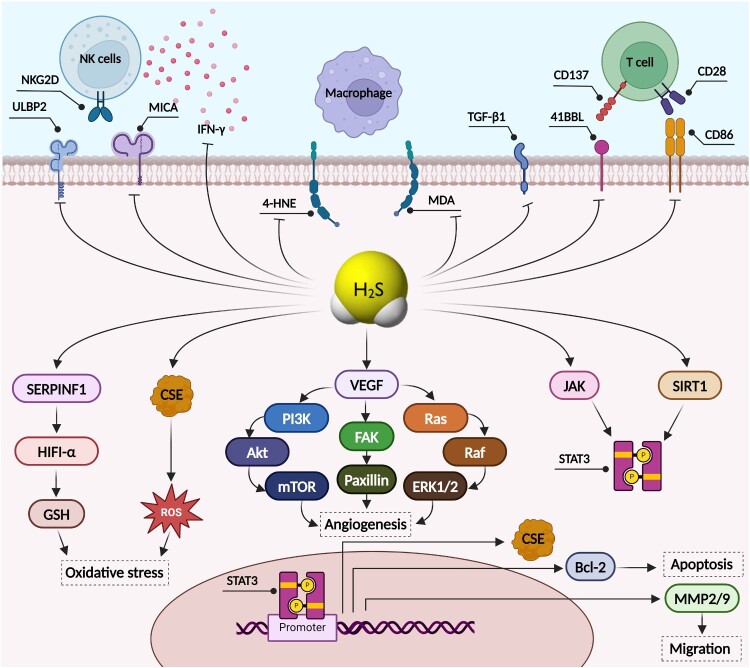
**Mechanistic role of H2S in breast cancer (BC) cells.** Implication of H_2_S in BC oncogenesis by controlling oxidative stress, angiogenesis, migration, apoptosis, and immunogenicity. 4-HNE: 4-Hydroxynonenal; Akt: Protein kinase B; ERK1/2: extracellular signal-regulated kinase 2; FAK: Focal adhesion kinase; GSH: Glutathione; HIFI-a: hypoxia-inducible factor-1 alpha; IFN-y: interferon gamma; JAK: Janus kinase; MDA: Malondialdehyde; MICA: MHC class I polypeptide – related sequence A; MMP2/9: matrix metalloproteinase-2/9; mTOR: mammalian target of rapamycin; PI3K: phosphoinositide 3-kinase; SERPINF1: serpin F1; SIRT1: Sirtuin-1; STAT3: Signal transducer and activator of transcription 3; TGF-β1: transforming growth factor beta-1; ULBP2: UL16-binding protein 2; VEGF: vascular endothelial growth factor.

H_2_S is involved not only in physiological regulation but also in many pathophysiological processes [14]. These pathophysiological processes include various forms of cancer [2,15,21,26–28]. H_2_S, when generated endogenously or when added to cancer cells at relatively low concentrations, can promote cancer progression by stimulating cancer cell growth, facilitating angiogenesis, and promoting resistance to chemotherapy (Figure 1) [20,21]. However, H_2_S, when added to cancer cells exogenously at higher concentrations, can also have antitumor effects by inducing apoptosis and inhibiting cancer cell proliferation by reducing DNA synthesis and arresting the cell cycle [16,29,30]. H_2_S donors were found to inhibit the PI3 K/AKT/mTOR, RAS/RAF/MEK/ERK, AKT/GSK-3β/β-catenin and EGFR/ERK/MMP-2 signaling pathways (Figure 1), which are key signal transduction pathways involved in the progression of different malignancies [31–34]. H_2_S donors protect lung cancer cells from nickel-induced epithelial–mesenchymal transition (EMT) and migration by impeding the TGF-β1/Smad2/Smad3 signaling pathway (Figure 1), whose upregulation mediates EMT and metastasis at late stages of tumorigenesis [33,35]. Conversely, inhibition of H_2_S-synthesizing enzymes either pharmacologically or by molecular inhibitors such as microRNAs (miRNAs) has been reported to counteract cancer progression and decrease cancer hallmarks [4,36–38]. Up to date, there is no clinical trial done to evaluate the role of H_2_S donor or inhibitors in cancer although some of H_2_S donors such as SG1002 is in clinical trial now for its therapeutic impact in heart failure patients (NCT01989208). Another H_2_S donor, GIC-1001, was also tested for its safety and bioavailability in healthy volunteers (NCT01738425). Interestingly, GIC-1001 was reported to be well tolerated, and showed a safety profile that is similar to that of placebo. Of note, GYY4137, a slow-releasing H_2_S donor, has been tested *in-vivo* on mice bearing leukemia xenograft and showed significant reduction in tumor growth [39]. On the other hand, H_2_S inhibitors didn’t reach clinical trials phase [40], yet despite the potent tumor growth inhibition in orthotopic BC-bearing nude mice [37]. Nevertheless, further optimization of pharmacokinetics and target specificity are still needed [40].

It should be stressed that the complex roles and effects of H_2_S in oncology depend on the concentration of H_2_S to which the cells are exposed and, at least in part, on the cell type and experimental model used [41,42]. For instance, a recent review revealed that tissues in which endogenous H_2_S levels are physiologically high (e.g. the brain and liver) tend to downregulate H_2_S biosynthesis after malignant transformation, while many tissues in which basal endogenous H_2_S levels are lower tend to upregulate various H_2_S-generating enzymes in cancer [28].

One of the substantial processes in which H_2_S is involved is cancer immunosurveillance [43,44]. In recent years, extensive research has been carried out to determine the role of H_2_S not only in the tumor microenvironment (TME) but also in immunomodulation in general and in the tumor immune microenvironment (TIME) in particular (Figure 1) [43]. Regulators of H_2_S-synthesizing machinery and H_2_S intracellular levels have been reported to modulate the TIME [15–17,21,45]. However, similar to its controversial role in tumorigenesis, H_2_S can act as both an immunosuppressive agent and an immunostimulatory agent [43,46]. For instance, CBS-derived H_2_S has been shown to exert immunosuppressive effects by protecting breast cancer (BC) cells from activated macrophage-generated reactive oxygen species (ROS) in macrophage-BC cell cocultures [47]. Moreover, in a study by Youness and colleagues in which BC cells were cocultured with natural killer (NK) cells, a decrease in either CBS-derived or CSE-derived H_2_S led to an increase in NK cell-mediated cytotoxity (Figure 1) [15]. On the other hand, treating melanoma-bearing mice with diallyl trisulfide (DATS), an H_2_S donor, inhibited tumor growth by modulating the TIME and increasing the recruitment of CD8+ T cells and dendritic cells (DCs) [48]. In addition, DATS treatment impeded the activity of myeloid-derived suppressor cells (MDSCs), which are involved in the promotion of tumor cell survival and immunotherapy resistance in patients with melanoma [48].

Many aspects of the complex role of H_2_S in cancer have been investigated over the last decade. However, although the cellular levels of H_2_S are clearly regulated both at the level of its production (i.e. enzymatic biosynthesis) and degradation (enzymatic and nonenzymatic) – less attention has been paid to the latter components. The current review, after a brief discussion of the regulation of the enzymes involved in H_2_S production, focuses on the present state of knowledge regarding the regulation and function of enzymes involved in H_2_S metabolism.

## 2. H_2_S-synthesizing enzymes in cancer

H_2_S is generated through nonenzymatic and enzymatic processes [49]. In the presence of reducing equivalents such as nicotinamide adenine dinucleotide phosphate (NADPH) and nicotinamide adenine dinucleotide (NADH), sulfane sulfur is reduced, and H_2_S is produced [50]. Additionally, persulfides, thiosulfate, and polysulfides, which are by products of amino acids metabolism that are found in the blood stream, can be chemically reduced in the presence of NADPH and NADH to H_2_S, particularly in hypoxic conditions [51]. These nonenzymatic processes of H_2_S synthesis are believed to be important in the recycling of biologically available sulfur. On the other hand, H_2_S is enzymatically produced via cystathionine-β-synthase (CBS), cystathionine-γ-lyase (CSE), 3-mercaptopyruvate sulfurtransferase (3-MST) [52] and cysteinyl-tRNA synthetase 2 (CARS2). In the following sections, the exact mechanistic synthesis pathway will be discussed. Further, the known involvement of these enzymes in tumorigenesis will be highlighted in detail.

### 2.1. Cystathionine β-synthetase (CBS)

In 1969, CBS was identified for the first time by Braunstein and colleagues. Later, the *CBS* gene was found to be located on chromosome 21. CBS is a polypeptide with a molecular weight of 63 kDa [53,54]. A shorter isozyme (≈48 kDa) was isolated from the liver; this enzyme lacks an allosteric regulatory site and has increased catalytic activity [55]. CBS is responsible for H_2_S production in various tissues, including the brain, liver and kidney [49]. Many pathophysiological events, including tumorigenesis, have been linked to CBS dysregulation [56,57]. Cancerous tissues of the colon [58], prostate [59], ovary [60], kidney [61], and BC [15] have shown higher expression levels of CBS than surrounding noncancerous tissues. A recent study showed that as mutations in the tumor suppressor genes *APC*, *SMAD4* and *TP53*, as well as the oncogene *KRAS* are introduced into human isogenic colonic epithelial cell organoids, the organoids assumed more proliferative and invasive properties and this was associated with a gradual upregulation of CBS at the stages of the initial mutations and truncated (i.e. constitutively over-activated) CBS as the mutations further accumulated [62]. High intra-tumoral levels of CBS have been linked to increased tumor growth and metastasis, as well as increased resistance to chemotherapy [58]. In contrast, brain tumor tissues and liver cancer tissues have been reported to have lower CBS expression profiles than corresponding noncancerous tissues [28,63].

### 2.2. Cystathionine-γ-lyase (CSE)

CSE protein is encoded by the cystathionine-gamma-ligase (*CTH*) gene on the short arm of chromosome 1 (1p31.1) and consists of 12 exons [64]. Another 11-exon transcription variant that lacks exon 5 has lower activity than the 12-exon variant [65]. CSE is a 405-residue polypeptide of 45 kDa molecular weight. Similar to CBS, the catalytic activity of CSE is also PLP-dependent. Besides, CSE depends on the amino acid L-cysteine as a precursor for H_2_S synthesis [64]. The role of CSE was first recognized in the cardiovascular and respiratory systems [49,66]. Endothelial cells and smooth muscle cells in blood vessels both synthesize significant amounts of H_2_S via CSE [67]. CSE is also reported as a significant H_2_S-synthesizing enzyme in the liver, kidney, pancreatic islets, and uterus [49].

CSE dysregulation was found to be associated with tumor progression. Overexpression of CSE has been reported in prostate [68], colon [62,69], liver [70], nasopharyngeal [71], skin [72], and breast [15] cancers. Pharmacological inhibition of CSE activity has been shown to inhibit the growth and proliferation of cancer cells, indicating its role in cancer development [36,37]. Notably, in BC and prostate cancer, metastatic lesions have shown greater expression levels of CSE than primary tumor lesions, emphasizing the particular association between CSE and the metastatic ability of neoplastic cells [68, 73]. CSE is involved in the synthesis of the potent antioxidant glutathione (GSH) [74,75]. Cancer cells with higher glutathione levels are better able to withstand the oxidative stresses associated with cancer progression and thus have a survival advantage [76]. Although selective CSE inhibitors are not commercially available yet. However, several non-coding RNAs have been reported to repress the expression of CSE in various solid malignancies such as microRNA-4317 [15], microRNA-939-5p [17], microRNA-193 [45] and microRNA-548 [45] as previously reviewed in [77].

### 2.3. 3-Mercaptopyruvate sulfurtransferase (3-MST)

Human 3-MST is another member of the H_2_S-synthesizing enzyme family [78]. It is encoded by the mercaptopyruvate sulfurtransferase (*MPST*) gene on the long arm of chromosome 22 in the subtelomeric region q13.1 [79]. The *MPST* gene is 5.6 kbp in length and contains only two exons [80]. The substrate of 3-MST is 3-mercaptopyruvate (3-MP), which is produced by the cysteine aminotransferase (CAT) enzyme from L-cysteine in the presence of α-ketoglutarate [49]. While CSE is mainly cytosolic and CBS is mainly cytosolic but can also be translocated to the mitochondria, 3-MST is present both in the cytosol and in mitochondria at comparable levels under physiological conditions [71]. Histologically, 3-MST is localized to, among other organs, the perivenous hepatocytes in the liver, the proximal tubular epithelium of the kidney and various cell types in the myocardium [49]. 3-MST can also assume a compensatory role in the brain in the absence of CBS enzyme [16,78]. 3-MST plays a critical role in the maintenance of cellular metabolism and bioenergetics [81,82]. In recent years, there has been growing interest in the role of 3-MST in cancer progression [83]. Several studies have shown that 3-MST is upregulated in various types of cancers, including colon cancer [36,62], brain gliomas [84], lung carcinoma [85], renal cancer [86], oral cancer [87], BC [88], and glioblastoma cell lines [89,90]. Although the exact mechanisms by which 3-MST promotes cancer progression are not fully understood, 3-MST is believed to be involved in several key cellular processes [83,90]. For instance, 3-MST has been reported to promote cancer cell survival by protecting cells from oxidative stress and by modulating the expression of genes involved in cell cycle regulation and apoptosis [91,92]. In addition, 3-MST has been linked to the regulation of cellular metabolism. A recent study by Santos and colleagues in breast cancer demonstrated that 3-MST supports cancer cell growth and proliferation by stimulating mitochondrial electron transport and ATP production [91].

### 2.4. Cysteinyl-tRNA synthetase 2 (CARS2)

Cysteinyl-tRNA synthetase 2 (CARS2) is a highly conserved enzyme in eukaryotic mitochondria encoded on the *CARS2* gene of the long arm of chromosome 13 at subtelomeric region q34 [93]. CARS2 is characterized by its distinctive catalytic domain responsible for the aminoacylation of tRNA molecules, and its catalytic activity involves the binding of cysteine and ATP, followed by the transfer of activated cysteine to the 3’ end of tRNA-(Cys), where it forms cysteinyl-tRNA [93]. In addition to its enzymatic function, CARS2 has been shown to interact with various protein partners involved in cellular processes such as mitochondrial biogenesis [94], stress response [95], and apoptosis regulation [95,96]. These interactions further underscore the multifaceted nature of CARS2 and its potential as a therapeutic target in cancers such as HCC [97], CRC [98], and basal-like breast cancer (BLBC) [99]. While H_2_S is primarily generated by the previously mentioned enzymatic pathways, recent studies have identified cysteinyl-tRNA synthetases, including CARS2, as alternative sources of H_2_S, particularly under physiological conditions [100]. The mechanism by which CARS2-mediated H_2_S production contributes to various aspects of cancer biology, including cell proliferation and migration, is poorly understood and requires further investigation [101].

## 3. H_2_S-catabolizing processes in cancer

### 3.1. Nonenzymatic H_2_S catabolism

**Figure 2.**
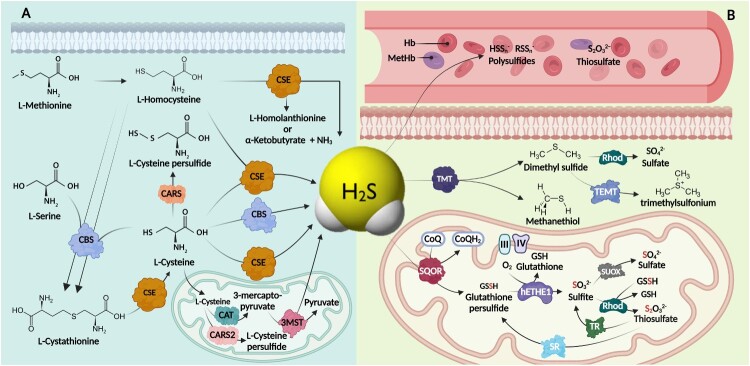
**H_2_S metabolic pathways.** (A). Anabolic pathways of H_2_S in the cytoplasm and mitochondrial matrix. (B). Enzymatic and nonenzymatic catabolic pathways of H_2_S either through oxidation or methylation, where oxidation occurs in the mitochondria enzymatically or in the blood nonenzymatically. CBS: cystathionine β synthase; CSE: cystathionine γ-lyase; 3MST: 3-mercaptopyruvate sulfurtransferase; CAT: cysteine aminotransferase; SQOR: sulfide quinone oxidoreductase; hETHE1: human ethylmalonic encephalopathy protein 1; Rhod: rhodanese; SUOX: sulfite oxidase; TR: thiosulfate reductase; SR: sulfur transferase; III: complex III; IV: complex IV; TMT: thiol S-methyltransferase; Hb: hemoglobin; MetHb: methemoglobin.

H_2_S is catabolized either nonenzymatically or enzymatically (Figure 2) [102]. In the circulation, one of the nonenzymatic catabolic pathways involves the conversion of H_2_S to polysulfide and thiosulfate by the heme iron of methemoglobin (MetHb), which is an oxidized form of Hb (ferrous is oxidized to ferric) that represents 1–3% of total Hb [103]. Furthermore, myoglobin and other globin species, such as neuroglobin, have been reported to be able to oxidize H_2_S into polysulfide and thiosulfate [104,105]. Likewise, H_2_S can undergo intracellular autooxidation under both aerobic and anaerobic conditions, leading to the production of polysulfide or thiosulfate, respectively, at physiological pH.

### 3.2. Sulfide oxidation unit (SOU)

Enzymatic H_2_S catabolism occurs substantially in the mitochondria and involves several oxidation pathways [102,106]. The sulfide oxidation unit (SOU) is a cluster of mitochondrial enzymes that are responsible for H_2_S metabolism [102,107]. It consists of (i) sulfide: quinone oxidoreductase (SQOR); (ii) human ethylmalonic encephalopathy protein 1 (hETHE1); (iii) rhodanese; and (iv) sulfite oxidase (SUOX). H_2_S is metabolized by SOU and undergoes a series of oxidation steps to finally produce thiosulfate and sulfate [102,106]. Thiosulfate was previously believed to be an inactive metabolite of H_2_S, but recent work has demonstrated that under various conditions, it can also be reconverted to H_2_S [102,108].

### 3.3. Sulfide: quinone oxidoreductase (SQOR)

Sulfide: quinone oxidoreductase (SQOR or SQR) is a mitochondrial membrane-bound enzyme encoded on the long arm of chromosome 15 with chromosomal location 15q21.1 [109]. SQOR was found to oxidize H_2_S in a process that eventually led to the generation of persulfide [102,110]. Together, the two redox active sites of SQOR are covalently attached to FAD and an adjacent pair of cysteine residues bridged by a chain of three sulfur atoms [111]. To oxidize H_2_S, SQOR produces uncharged sulfane sulfur (S^0^) with the help of coenzyme Q (CoQ), which acts as an electron acceptor that subsequently drives the electron transport chain (ETC) in its reduced form [102]. Then, SQOR transfers the sulfane sulfur to glutathione (GSH) or to any other sulfur acceptor, such as sulfite (SO_3_^2−^), cysteine, or homocysteine (Figure 2(B)).

There appears to be a significant correlation between SQOR overexpression and cancer progression. In fact, it has been described as a ‘respiratory shield against H_2_S poisoning’ in colorectal cancer (CRC) [112]. Libiad and colleagues reported that SQOR is overexpressed in six different CRC cell lines compared to human colonic epithelial cells, which represent a nonmalignant colon cell line. Additionally, out of seven clinical tissues collected from CRC patients, five have shown a significant increase in the expression level of SQOR at the protein level compared to the tissues collected from the normal margin [112].

As the first-step mediator of H_2_S mitochondrial metabolism and later uses H_2_S as an electron source for the mitochondrial electron transport chain, SQOR has been hypothesized to be the originator of H_2_S overexpression in oral squamous cell carcinoma (OSCC) [87]. Interestingly, in the same study by Ascenção, K., et al., they showed that when mutations in *APC*, *SMAD4, TP53*, and *KRAS* were introduced into colonic epithelial cell, a gradual upregulation of SQOR in response to the initial mutations, but as the mutations further accumulated, SQOR levels became downregulated, and at it was only at these later stages of the colonic carcinogenesis process where the organoids contained significantly higher levels of H_2_S than normal colonic epithelial cells [62]. This pattern was different from the situation with ETHE1 and TST (see below), which remained upregulated in the entire set of colonic organoids accumulating sequential mutations. Very limited data are available regarding SQOR expression in other cancer types [110].

### 3.4. Human ethylmalonic encephalopathy protein 1 (hETHE1)

Human ethylmalonic encephalopathy protein 1 (hETHE1) is also called sulfur dioxygenase (SDO) and persulfide dioxygenase (PDO) [113]. It is encoded by a gene located on chromosome 19's long arm at 19q13.31. The enzyme is located in the mitochondrial matrix [114,115]. hETHE1 catalyzes the synthesis of sulfites (SO_3_^2−^) from persulfide compounds. Since glutathione persulfide (GSSH) is a substantial product of SQOR under physiological conditions, hETHE1 uses GSSH to synthesize sulfite (SO_3_^2−^) and glutathione (GSH) (Figure 2(B)) [102,116,117].

Dysregulation of hETHE1 has been demonstrated in several pathophysiological conditions. For instance, one of the most well-known diseases related to *hETHE1* gene mutation is ethylmalonic encephalopathy (EE) [118]. It is a rare genetic disorder that is inherited in an autosomal recessive pattern. EE is characterized by defects in the brain, nerves, peripheral blood vessels, and gastrointestinal tract, as well as the presence of necrotic lesions in the deep gray matter of the brain, hemorrhagic diarrhea, neurodevelopmental delay, petechiae and acrocyanosis [119,120]. These symptoms manifest in the first decade of life. hETHE1 was found to be upregulated in colorectal cancer [112,118,121]. Ozluk and colleagues reported that ETHE1 expression was twofold greater in colorectal adenocarcinoma than in benign colonic epithelium [118]. Further, Ascenção, K., et al., reported gradual upregulation of ETHE1 in colonic epithelial cell organoids upon mutating *APC*, *SMAD4*, *TP53*, and *KRAS* [62].

### 3.5. Rhodanese

Rhodanese, also called thiosulfate transferase (TST), is the third enzyme of the SOU machinery and is encoded on chromosome 22 [110]. Rhodanese utilizes sulfite (SO_3_^2−^) as a co-substrate, yielding thiosulfate (S_2_O_3_^2−^), with the second sulfur coming from GSSH [51]. Thiosulfate, in turn, can be used in H_2_S anabolism by 3-MST and rhodanese [51,102]. Dysregulation of rhodanese has been demonstrated in various diseases; however, the functional role of this dysregulation has not been elucidated. Low expression levels of rhodanese have been found in liver biopsies from patients with Leber's hereditary optic atrophy [122]. No relationship between rhodanese expression and tumor development has been reported. Although rhodanese was found to be overexpressed in five out of seven CRC patients, this upregulation pattern was not observed in the CRC cell line compared to the nonmalignant human colonic epithelial cell line [112]. However, a study by Ascenção, K., et al., has reported gradual upregulation of rhodanese in organoids of human isogenic colonic epithelial cell that had mutations in oncogene and tumor suppressor genes [62].

### 3.6. Sulfite oxidase (SUOX/SO)

Sulfite oxidase (SUOX) is an enzyme encoded on the long arm of chromosome 12 at location 12q13.2 [115]. In parallel to rhodanese, SUOX exerts its catalytic effect on the catabolic product of hETHE1 [51]. Nonetheless, unlike rhodanese, SUOX catalyzes the synthesis of sulfates (SO_4_^2−^) from sulfite (SO_3_^2−^) obtained from hETHE1 (Figure 2(B)) [102]. SUOX dysregulation is believed to be a potential contributor to various diseases. Isolated sulfite oxidase deficiency (ISOD), which is caused by a mutation in the *SUOX* gene, is a life-threatening autosomal recessive inherited metabolic disorder [123]. Patients with ISOD have a defect in the conversion of sulfite (SO_3_^2−^) into sulfate (SO_4_^2−^) in the metabolism of sulfur-containing amino acids, leading to an increase in sulfite (SO_3_^2−^) levels, which in turn results in neurotoxic effects. The onset of SUOX deficiency symptoms usually occurs in the early infantile or neonatal period, and long-term effective treatment is unavailable [123]. The symptoms are characterized by neurological impairment, such as therapy-resistant seizures, developmental delay, psychomotor retardation, and microcephaly [123–125]. Moreover, alterations in the SUOX expression level are also linked to the progression of many malignant tumors. A study performed by Yano and colleagues on 98 patients with advanced gastric cancer demonstrated that low expression of SUOX could be a prognostic biomarker in gastric cancer. Interestingly, patients who had low SUOX expression were found to have a poorer prognosis and lower overall survival than patients who had high SUOX expression [126]. On the other hand, high SUOX expression was linked to postoperative recurrence in patients with prostate cancer [127]. Kurose and colleagues studied 97 patients who underwent radical prostatectomy and reported that patients who had SUOX overexpression were more susceptible to prostate cancer recurrence after prostatectomy than patients with low SUOX expression [127]. Additionally, patients with hepatocellular carcinoma and tongue cancer showed decreased SUOX expression with disease progression and infiltration.

### 3.7. Cytochrome c oxidase (CcO)

A less established route of H_2_S catabolism is the reaction with iron-heme species such as CcO [103,128]. CcO, also known as complex IV, is an enzyme located in the inner membrane of mitochondria and is involved in the mitochondrial respiratory chain [129]. Structurally, CcO is composed of different subunits: ≈ 8–11 are nuclear-encoded subunits, and three mitochondrial-encoded subunits are assembled from the assembled proteins. CcO contains copper and heme groups in its active site, making it potentially linked to H_2_S metabolism [130]. At low H_2_S concentrations, CcO acts as an electron donor to the ETC, stimulating ATP production and oxygen consumption through the sulfide oxidation pathway. This is achieved via the transfer of electrons from cytochrome c to oxygen, the terminal electron acceptor, which produces water molecules and a proton gradient [131]. Despite substantial efforts to understand the crosstalk between H_2_S and CcO, a validated theory describing the impact of CcO on H_2_S is currently lacking [130]. Yong and Searcy reported that chicken liver mitochondria consumed oxygen at a relatively high rate when supplied with low concentrations of H_2_S, which was coupled with increased ATP synthesis [131,132]. However, at high H_2_S concentrations, CcO was inhibited through the binding of H_2_S to the copper center in the active site of CcO [131]. In this context, low concentrations of H_2_S stimulate bioenergetics through oxidative phosphorylation, whereas high H_2_S concentrations cause toxic inhibitory effects on the CcO enzyme. Therefore, the effect of H_2_S on CcO is believed to be concentration dependent.

CcO deficiency is a genetic disorder that can be life-threatening, as deficient patients frequently do not overpass childhood [133]. Symptoms of CcO deficiency manifest after the age of 2 years and in mild cases at adolescence and include hypertrophic cardiomyopathy, myopathy, hypotonia, encephalomyopathy, Leigh syndrome and lactic acidosis, which can cause nausea and irregular heartbeats and can be life threatening [133]. Moreover, CcO dysregulation is linked to cancer progression. Zhang and colleagues reported that CcO expression was upregulated in colorectal carcinoma. This overexpression was confirmed at the genomic and proteomic levels. As a result, they suggested that the upregulation of CcO plays a vital role in colorectal carcinogenesis [134]. The potential role of the CcO system in the function of various cancer cells requires further investigation.

### 3.8. Thiol S-methyltransferase (TMT)

Along with its catabolism by oxidation, H_2_S can also be catabolized via methylation pathways [107]. Unlike the oxidation pathway, H_2_S methylation occurs mainly in the cytoplasm [135]. Notably, methylation is deemed less of a contributor to H_2_S detoxification than oxidation since it only catabolizes small quantities of H_2_S under physiological conditions [136,137]. Thiol S-methyltransferase (TMT) is the main enzyme responsible for H_2_S methylation (Figure 2) [136]. This process results in the production of methanethiol (CH_3_SH), dimethyl sulfide, or both in the cytoplasm [135,136]. DMS can be further oxidized by rhodanese to thiocyanate (SCN⁻) and (SO_4_^2−^)[137]. The potential role of the TMT system in cancer requires further investigation.

### 3.9. Thioether S-methyltransferase (TEMT)

Thioether S-methyltransferase (TEMT) is another methyltransferase enzyme that acts on various thioethers, leading to the generation of their respective sulfonium ions [138]. Upon binding to dimethyl sulfide, TEMT is converted to trimethylsulfonium (TMS), which is subsequently excreted in the urine [138,139]. Intriguingly, TMS has shown clinical potential as a biomarker for the level of endogenous H_2_S produced. In subjects with Down syndrome who overexpress CBS and H_2_S, the urinary levels of TMS are more sensitive to endogenous H_2_S than the traditional biomarker thiosulfate [140]. The potential role of the TEMT system in cancer requires further investigation.

## 4. Implications and expert opinion

Understanding H_2_S metabolism is promising for future translational applications. To date, most studies have focused on H_2_S-synthesizing enzymes rather than on H_2_S-metabolizing enzymes, especially in oncological contexts. Nevertheless, there are several studies – including a recent study where various relevant mutations in the tumor suppressor genes *APC*, *SMAD4* and *TP53*, as well as the oncogene *KRAS* are introduced into human isogenic colonic epithelial cell organoids [62], where the upregulation of various H_2_S-producing enzymes (CBS, CSE, 3-MST) is also associated with an upregulation of various H_2_S-degrading enzymes (SQOR, ETHE1, rhodanese), but as the organoids accumulate multiple mutations, the expression of SQOR decreases. Interestingly, it is only these organoids (where multiple H_2_S producing enzymes are upregulated, but SQOR is downregulated), where the ‘ambient’ H_2_S levels show an increase. These data may indicate that there may be an initial, perhaps compensatory upregulation of H_2_S-degrading enzymes in early stages of carcinogenesis/tumor progression, where the increased H_2_S generation may be compensated by increased H_2_S degradation. Interestingly, a similar, perhaps also compensatory upregulation of H_2_S degrading enzymes is also noticeable in Down syndrome models, where CBS and 3-MST are upregulated, but this is also accompanied by an upregulation of various H_2_S catabolizing enzymes [141–143]. A simultaneous increase in H_2_S production and H_2_S degradation, however, does not mean that the ‘reactive sulfur system’ is not affected: one would expect that under such conditions, the cells and tissues accumulate more thiosulfate and more polysulfides as well. These species may have their own biological effects, for example, increased polysulfides may, in turn, mediate increased protein persulfidation reactions. At the later, most aggressive stage of cancer, however, some of the H_2_S-degradation systems may become downregulated. As an example, in the colonic epithelial cell organoid study mentioned above, SQOR (but not rhodanese or ETHE1) becomes downregulated. This combination of processes, in turn, can result in a (further) elevation of cellular H_2_S levels.

Taken together, the evidence reviewed in the current article indicates that – in addition to H_2_S biosynthetic enzymes – further attention should be paid to the regulation of various H_2_S degradation systems in various forms of cancer, and these two system groups should be integrated when formulating mechanistic hypotheses on the role of reactive sulfur species in the pathogenesis of various forms of cancer. While the regulation of CBS, CSE, 3-MST (and to a lesser degree CARS2) in various forms of cancer has been studied intensively, the regulation of the various H_2_S degradation pathways remains to be investigated. For example, future work should pay more attention to the effect of SOU, rhodanese, ETHE1 or CcO dysregulation on H_2_S production in different tumors. Also, correlation studies, using various publicly available cancer databases, should be conducted to test if correlations exist between the regulation of various H_2_S degradation pathways and clinical outcomes in various forms of cancer (such studies, with respect to H_2_S synthesizing enzymes, already exist and indicate that in various forms of cancer, e.g. ovarian or colon cancer, upregulation of CBS correlates with a poor clinical prognosis).

## 5. Conclusion and future directions

How, then, could the present (and future, accumulating) information on H_2_S degradation pathways and cancer could be translated into future clinical/translational concepts? The biology of H_2_S is unique, due to the characteristic bell-shaped concentration-response curve of this gasotransmitter, whereby cells prefer a certain intermediate concentration of H_2_S, and marked decreases, as well as marked increases from this preferred level are both detrimental to cell viability/cell survival. This is also true for cancer cells, which upregulate their ambient H_2_S and polysulfide levels, which serves their increased metabolic demands, increased proliferation, and tumor angiogenesis. Thus, approaches that decrease H_2_S levels within tumor cells (e.g. via inhibition of H_2_S biosynthetic enzymes) and approaches that increase H_2_S levels in tumors to higher (cytotoxic) levels (e.g. via additional H_2_S donation) can lead to decreased tumor cell metabolism, suppression of tumor cell proliferation and enhancement of tumor cell elimination via cytotoxic/cytostatic agents or the immune system. When we apply the same concept to H_2_S degradation enzymes, it can be argued that stimulation/forced overexpression of H_2_S degradation enzymes may also lead to decreased intratumoral H_2_S levels (possibly decreasing tumor cell viability), but also inhibition of H_2_S degradation enzyme activity (which, in turn, may elevate H_2_S levels in the tumor to levels that are supraoptimal, i.e. start to become detrimental) may be therapeutically useful. Further work remains to be conducted to test these possibilities. One limitation for this research direction is that most of the H_2_S degradation enzymes do not have potent or selective pharmacological inhibitors, i.e. the currently available experimental approaches will have to focus on forced overexpression and/or silencing of these enzymes. It should also be determined which H_2_S degradation enzymes are most relevant for which type of tumor since literature didn’t answer this explicit question so far. Given the known literature, any hypothesis making for specific focus on a particular enzyme for future studies, will be biased. Instead, logical experimental design to allow better determination of workhorse enzymes are more effective. Indeed, more than decade ago, literature showed that, CBS not CSE, is responsible for colon cancer progression [38]. Yet, after including the 3D nature of organoid and involving H_2_S-catabolizing enzymes as well, CSE and other key player seemed to be also participating in tumorgenesis [62]. Therefore, such a question is supremely crucial because modulation of multiple enzymes/pathways is translationally more difficult than targeting a single enzyme/pathway.

Taken together, significant preclinical work remains to be conducted in order to understand the role of H_2_S in oncogenesis and tumor progression, and this work must include further investigation to better understand the intricate interplay between H_2_S-synthesizing and H_2_S-catabolizing enzymes in various forms of cancer. The ultimate goal of these efforts remains the formulation of personalized approaches to fight cancer development and progression based around the modulation of intratumoral H_2_S levels.

## Data Availability

All data and resources used in the paper have been cited and indicated.
